# The Demography of Traumatic Brachial Plexus Avulsion Injuries

**DOI:** 10.7759/cureus.25626

**Published:** 2022-06-03

**Authors:** Ramachandran Viswamadesh, Subashini Rajendiran, Arvind Maharaj Pakiri Maheswaran, Karthikeyan Gomathinayagam

**Affiliations:** 1 Plastic and Reconstructive Surgery, Cancer Institute Adayar, Chennai, IND; 2 Hand Surgery, Sri Ramachandra Institute of Higher Education and Research, Chennai, IND; 3 Plastic and Reconstructive Surgery, SRM Medical College Hospital and Research Centre, Chennai, IND; 4 Plastic and Reconstructive Surgery, Tamil Nadu Government Multisuperspeciality Hospital, Omandurar Estate, Chennai, IND

**Keywords:** elbow flexion, shoulder abduction, nerve transfer, avulsion injuries, brachial plexus injuries

## Abstract

Background and objective

Brachial plexus injuries more commonly affect the younger generation who constitute the productive workforce. The patients who sustain avulsion injuries of the brachial plexus are more often involved in high-velocity accidents. The avulsion injuries are surgically managed by nerve transfers. This study aimed to evaluate the demography of brachial plexus avulsion injuries.

Materials and methods

This retrospective study was conducted in January 2013 and included 21 patients treated from January 2007 to December 2011.

Results

Of the 21 patients, 20 were male and the most commonly affected patients were in the age group of 21-30 years. The mean age of the affected patients was 27.24 years. Six of the patients had pan palsy (C5-8 and T1), nine had C5-7 injury, and six had C5-6 injury. Twenty patients underwent spinal accessory to suprascapular nerve transfer, nine patients underwent ulnar nerve fascicle to nerve to biceps branch transfer, and one patient underwent intercostal nerve to musculocutaneous nerve transfer. Of note, 40% of the patients regained more than M3 power for abduction and external rotation of the shoulder, and 30% of the patients regained more than M3 power for elbow function.

Conclusions

Road traffic accidents are the most common cause of brachial plexus injuries. Nerve transfers for shoulder and elbow function play a significant role in improving the function of the upper extremity.

## Introduction

Brachial plexus injuries are on the rise due to an increase in high-velocity motor accidents and they more commonly involve the younger age groups. The main causes of brachial plexus palsies are traction, due to extreme movements, and heavy impact [1]. Closed brachial plexus injuries are more common than those with open skin injuries. Apart from trauma, the other causes of closed brachial plexus injuries are compression, tumor, and infections. Brachial plexus injuries with open skin wounds occur due to gunshot injuries or assault with sharp objects. Adult brachial plexus injuries result in profound functional deficits, debilitating pain, substantial mental health implications, and extensive economic impacts [2].

The management involves thorough examination and evaluation by nerve conduction study, electromyography, and imaging. Avulsion of the spinal roots from the spinal cord versus stretch or rupture to the plexus is differentiated by clinical examination and investigations. The management protocol is established and then carried out. Nerve grafting and neurotization (the transposition of a functioning nerve to the distal stump of an injured or divided nerve to provide axons for regeneration) now offer greater opportunity for a return to effective function [3]. This study aims to evaluate the outcomes related to shoulder and elbow functions after multiple nerve transfers in brachial plexus avulsion injuries.

## Materials and methods

This retrospective study was conducted in January 2013 at the Institute for Research and Rehabilitation of Hand and the Department of Plastic Surgery at the Government Stanley Hospital, Chennai, and included patients treated from January 2007 to December 2011. Patients who had brachial plexus root avulsion injuries and underwent nerve transfer surgeries were included. The patients who had other types of brachial plexus injuries, associated major injuries, associated skeletal injuries of the same limb, and birth brachial plexus palsies were excluded. Based on the hospital records, of the 134 patients with brachial plexus injuries, 21 patients fulfilled the inclusion and exclusion criteria.

The following data were gathered from the case sheets for analysis:

1. Age

2. Gender

3. Occupation

4. Level of injury

5. Side of injury

6. Mode of injuries

7. Nerve transfer surgeries done

Sixteen of the 21 patients responded to phone calls and presented for outcome evaluation of the surgeries. As for the five remaining patients, results were obtained from the records of their final visit.

## Results

**Figure 1:**
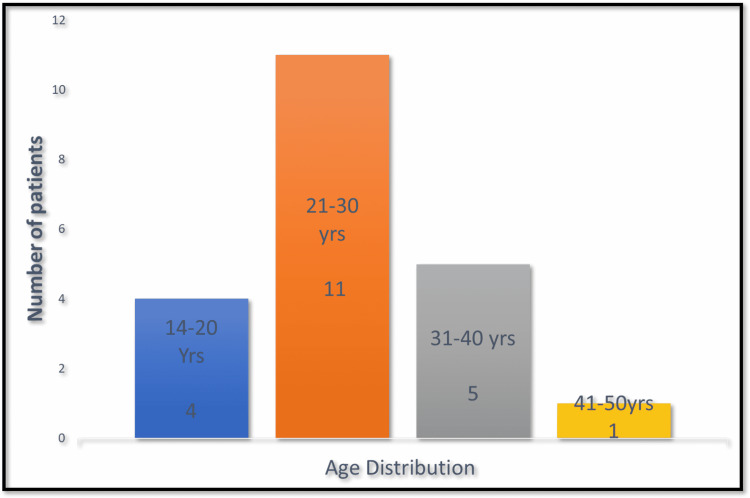
Analysis of the age distribution of patients

Of the total 21 patients, there were 20 males and one female. Age group analysis showed that most of the patients were in the age group of 21-30 years (Figure 1).

**Figure 2:**
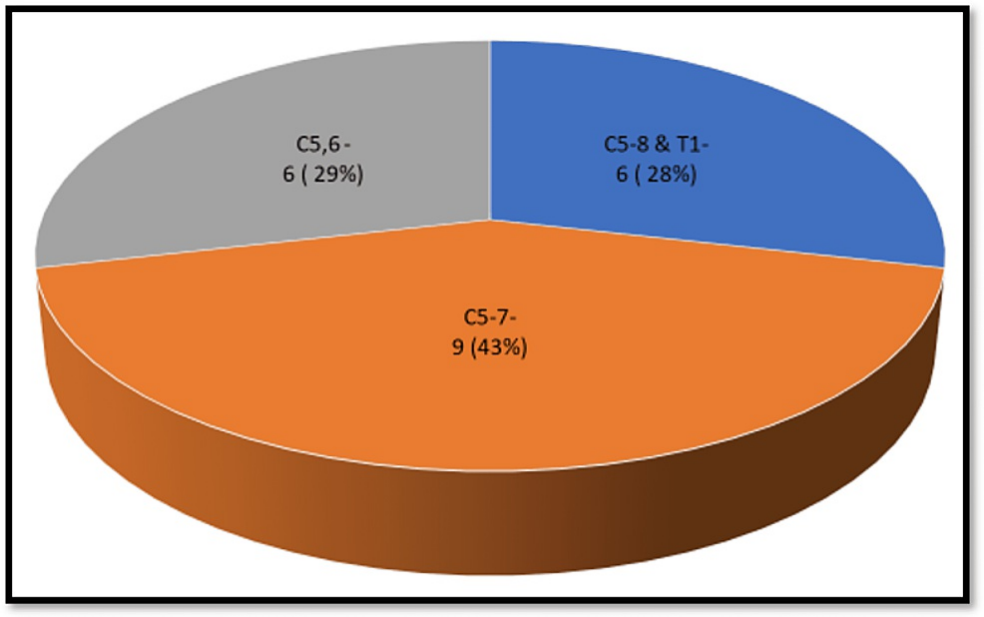
Analysis of the level of injury

The mean age of the affected patients was 27.24 years. Sixteen of the affected patients were manual laborers, four were students, and one was a sedentary worker. Six of the patients had pan palsy (C5-8 and T1), nine had C5-7 injury, and six had C5-6 injury (Figure 2). The right side was affected in 11 patients and the left side was affected in 10 patients.

**Figure 3:**
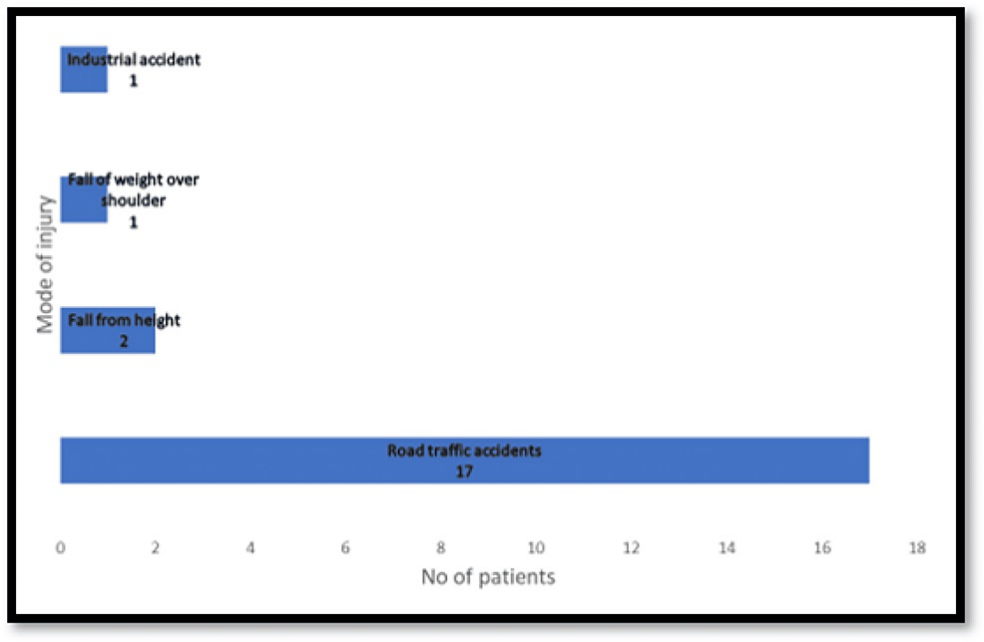
Analysis of the mode of injury

Seventeen of the patients sustained injury due to road traffic accidents, two patients sustained injury due to a fall from a height, one patient had an injury due to the fall of a heavy weight over the shoulder, and the remaining one patient had an injury due to an industrial accident (Figure 3).

**Figure 4:**
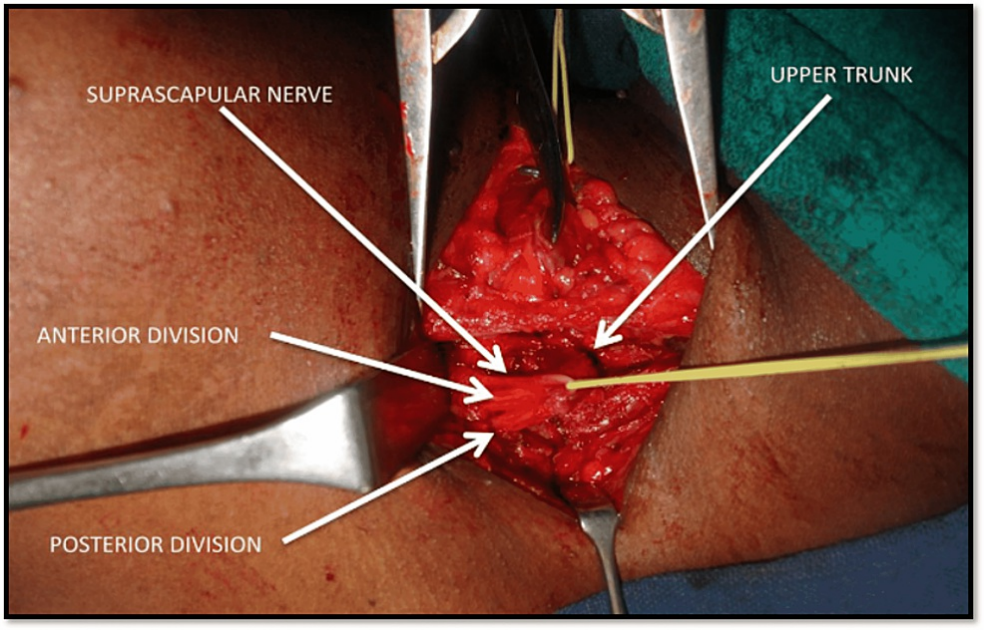
Exploration in a brachial plexus injury

**Figure 5:**
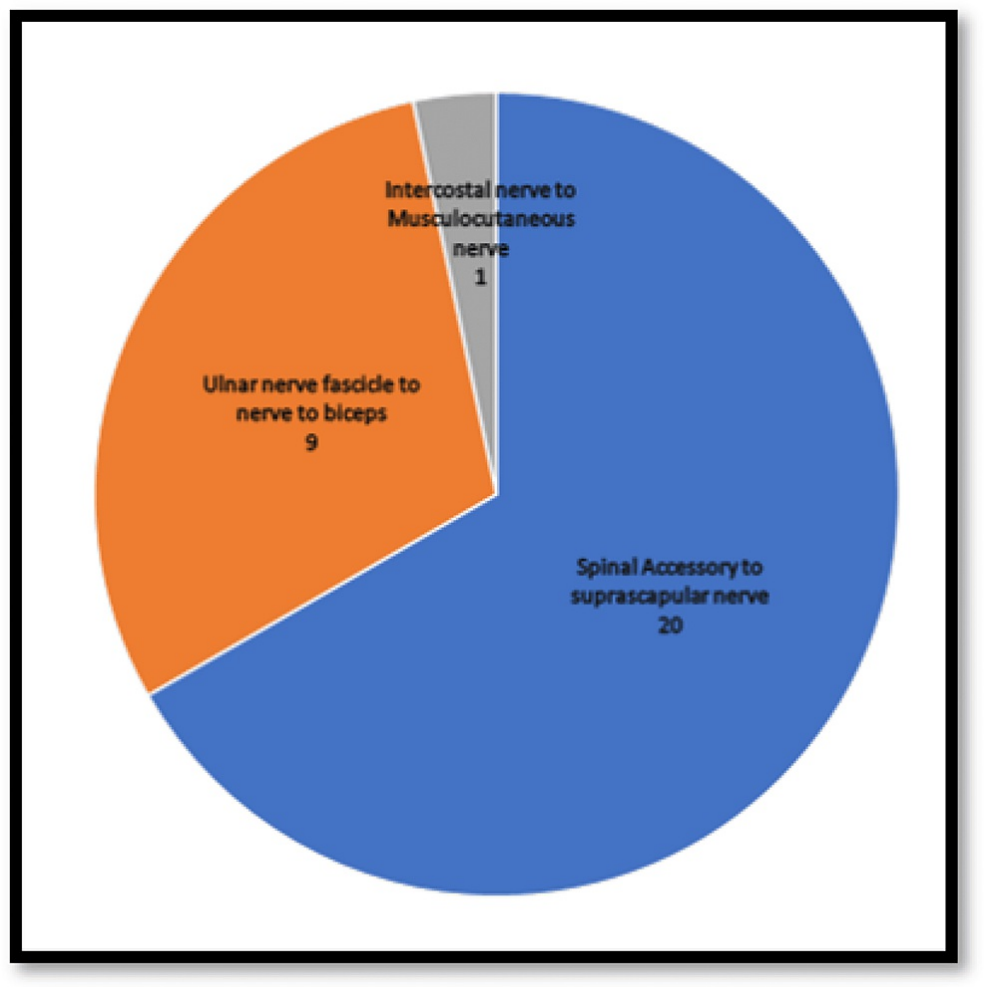
Nerve transfer surgeries done

All 21 patients underwent exploration of the brachial plexus (Figure 4); 20 patients underwent spinal accessory to suprascapular nerve transfer, nine patients underwent ulnar nerve fascicle to nerve to biceps transfer, and one patient underwent intercostal nerve to musculocutaneous nerve transfer (Figure 5).

The spinal accessory nerve to suprascapular nerve transfer had a failure rate of 25% (5/20 patients had less than 30 degrees of shoulder abduction) and a mean range of shoulder abduction of 48 degrees; 40% of the patients regained more than M3 power for supraspinatus.

**Figure 6:**
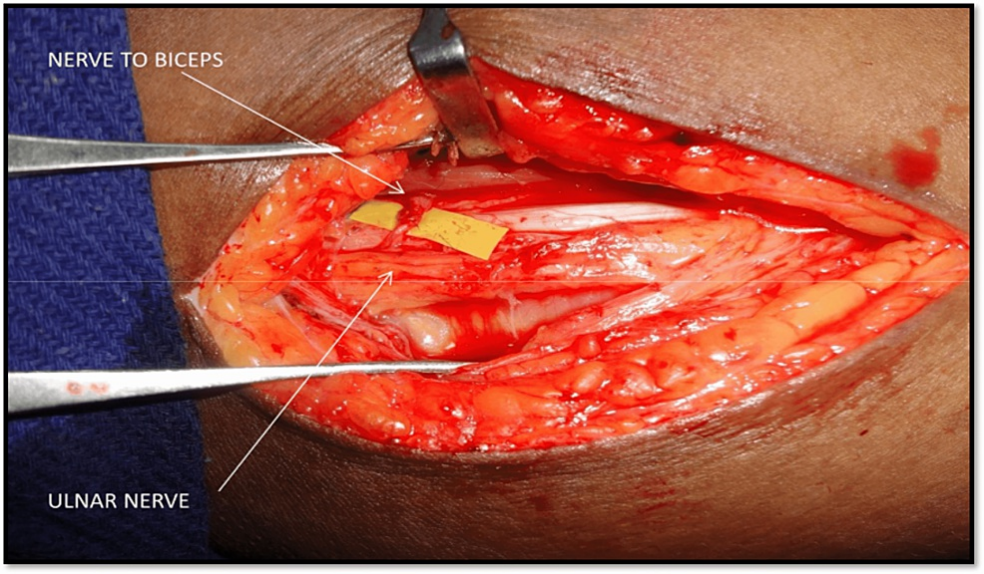
Coaptation of ulnar nerve fascicle to nerve to biceps

Of the nine patients who underwent ulnar nerve fascicle to nerve to biceps transfer (Figure 6), one patient did not have any recovery in elbow flexion. The mean range of elbow flexion was 86.25 degrees. Of note, 30% of the patients regained more than or equal to M3 power for biceps.

The procedures performed and their outcomes are summarized in Table 1.

**Table 1:** Data of patients with nerve transfers done and their outcomes MRC: Medical Research Council; SA-SSN: spinal accessory nerve to suprascapular nerve transfer; OB-1: ulnar nerve fascicle to branch to biceps transfer; ICN-MCN: intercostal nerves to musculocutaneous nerve transfer

Patient	Roots involved	Nerve transfer for shoulder	Range of shoulder abduction in degrees	MRC grade - supraspinatus	Nerve transfer for elbow flexion	Range of elbow flexion in degrees	MRC grade - biceps
1	C5, C6, C7	SA-SSN	0-40	M3	OB-1	0-90	M3
2	C5, C6	SA-SSN	0-40	M3	OB-1	0-120	M4
3	C5, C6, C7	SA-SSN	NIL	M0	-	-	-
4	C5-8, T1	SA-SSN	0-40	M3	-	-	-
5	C5-8, T1	SA-SSN	NIL	M0	-	-	-
6	C5-8, T1	-	NIL	-	ICN-MCN	NIL	M0
7	C5, C6, C7	SA-SSN	0-30	M2	OB-1	NIL	M0
8	C5, C6, C7	SA-SSN	0-90	M3	OB-1	0-70	M3
9	C5, C6, C7	SA-SSN	0-40	M3	OB-1	0-70	M3
10	C5, C6	SA-SSN	0-40	M3	OB-1	0-70	M3
11	C5, C6, C7	SA-SSN	0-90	M4	-	-	-
12	C5-8, T1	SA-SSN	NIL	M0	-	-	-
13	C5, C6, C7	SA-SSN	0-40	M2	-	-	-
14	C5, C6, C7	SA-SSN	0-40	M3	OB-1	0-90	M3
15	C5-8, T1	SA-SSN	0-30	M2	-	-	-
16	C5-8, T1	SA-SSN	NIL	M0	-	-	-
17	C5, C6	SA-SSN	0-90	M4	-	-	-
18	C5-8, T1	SA-SSN	0-30	M2	-	-	-
19	C5, C6	SA-SSN	0-40	M3	OB-1	-	M2
20	C5, C6, C7	SA-SSN	0-40	M3	OB-1	0-90	M3
21	C5, C6, C7	SA-SSN	NIL	M0	-	-	-

## Discussion

In our study, 95.24% of males and 4.76% of females were affected by brachial plexus injuries. This is similar to the findings of the study by Faglioni et al. [4], which reported 94.6% of males and 5.45% of females being affected. Our findings are also comparable to the 93% pooled prevalence of male patients from seven studies in the systematic review and meta-analysis by Kaiser et al. [5].

The age of patients in our study ranged from 19 to 45 years. Though the age range was 14-63 years in the study by Midha [6], the mean age was 29 years, which is comparable to the mean age of 27.24 years in our study. Also, it is comparable to the mean age of 28.4 years in the study by Faglioni et al. [4]. Road traffic accidents were the most common cause of injuries in our study (81%), which is similar to many other studies [4,5,6].

Moran et al. state that root avulsions are present in 75% of cases of the supraclavicular lesion and multiple root avulsions have become more frequent over the past 25 years [7]. The major problem with avulsions is the lack of proximal intraplexus donors in continuity with the spinal cord [8]. As anatomical restoration of brachial plexus is not possible in avulsion injuries, neurotization procedures are usually performed.

The common nerve transfers for shoulder reanimation include the spinal accessory nerve to the suprascapular nerve and the triceps branch of the radial nerve to the axillary nerve [2]. The spinal accessory nerve to suprascapular nerve transfer had a failure rate of 25% (5/20 patients had less than 30 degrees of shoulder abduction) and a mean range of shoulder abduction of 48 degrees. Bertelli et al. reported a 25% failure rate and a mean range of shoulder abduction of 45 degrees in the initial period of their study [9]. They state in their study that after the introduction of exploration of the suprascapular nerve till the suprascapular fossa in 2005, the failure rate fell to 9% and they achieved a mean range of shoulder abduction recovery of 62% [9]. The Medical Research Council (MRC) scale grading of recovery of shoulder abduction varies considerably in the literature. Gillis et al. [10] have reported 35% of patients having more than or equal to M3 and Malessy et al. [11] have reported 17% having the same. In our study, the MRC scale grading of more than or equal to M3 was seen in 40% of patients.

To restore elbow flexion, nerve branches to the biceps and/or brachialis from the musculocutaneous nerve were targeted [2]. Oberlin et al. have described two patients who did not have biceps recovery following biceps reinnervation using ulnar nerve fascicles in 29 patients [12]. In our series, one of the nine patients did not have biceps recovery. The mean average elbow flexion obtained in our study was 86.25 degrees. de Azevedo et al. have reported a mean average elbow flexion of 100.2 degrees [13]; 77.80% and 90% regained more than or equal to M3 MRC grade of biceps in the studies done by de Azevedo et al. [13] and Garg et al. [14] respectively. In our study, only 30% had a recovery of more than or equal to M3 MRC grade of elbow flexion.

More than 50% of the patients did not turn up for nerve transfer for elbow surgery after their procedure for the shoulder, due to various reasons: a) 70% of the defaulters cited financial reasons, b) 20% did not show up due to social reasons, and c) 10% due to their dissatisfaction with the results of the first surgery, which had failed to meet their expectations.

Our study has a few limitations. Firstly, this was a retrospective study and selection bias of favorable patients for surgery cannot be ruled out; the second limitation involves the varied interval between injury and the surgery. Finally, a few patients had less than one and a half years of follow-up, thereby influencing the outcome percentage. We believe that a prospective study adhering to a uniform timeframe for intervention and outcome analysis will provide us with a better understanding of the subject.

## Conclusions

Brachial plexus injuries are more commonly caused by high-velocity road traffic accidents. Hence, measures to prevent road traffic accidents should be implemented. Primary healthcare providers need to be educated about the recent advances in the treatment of brachial plexus injury and the favorable outcomes following timely nerve transfers so that patients are brought to the tertiary care center without delay. Nerve transfers increase the function of the upper extremity. Lastly, the patients need to be thoroughly enlightened about their problems and the planned surgeries, as well as the expected outcomes and the duration till the results manifest. The patients should also be provided with counseling and rehabilitation support. This multidisciplinary approach will contribute significantly to improving the outcomes of brachial plexus injuries.
